# A Case Report of Concurrent Cryptococcal and Tuberculous Meningitis in an Immunosuppressed Renal Transplant Patient

**DOI:** 10.7759/cureus.31012

**Published:** 2022-11-02

**Authors:** Linda Barasa, Ahmed Sokwala, Felix Riunga, Dilraj S Sokhi

**Affiliations:** 1 Medicine, Aga Khan University Hospital, Nairobi, KEN

**Keywords:** africa, immunosuppression, tuberculous meningitis, cryptococcal meningitis, renal transplant

## Abstract

Infections after renal transplant are a common cause of morbidity and are commonly due to Cytomegalovirus (CMV), *Cryptococcus*, *Mycobacterium tuberculosis*, and *Aspergillus*. Concurrent infections with both cryptococcal and tuberculous aetiologies are rare within the central nervous system (CNS). We present a case of a 67-year-old male patient who presented with three weeks of headaches, confusion, unsteady gait, and seizures. He had type 2 diabetes mellitus and hypertension. He had a kidney transplant three years prior and was on three immunosuppressive agents. He was HIV-negative. He was evaluated and found to have cryptococcal meningitis and received appropriate treatment with liposomal amphotericin B, flucytosine, and serial lumbar punctures. He also had treatment for CMV viremia with valganciclovir. Three weeks later, after an initial good clinical response, he deteriorated with worsening confusion and persistent seizures. We re-evaluated him and found him to have brain imaging suggestive of tuberculosis. We started him on anti-tuberculous medication, and he improved significantly and was alert and seizure free at discharge home one month later. This case highlights that concurrent CNS infections with cryptococcus and tuberculosis do occur especially in patients who are severely immunosuppressed such as after a renal transplant. Failure to improve while on treatment for one CNS opportunistic infection should prompt one to investigate for other concurrent causes.

## Introduction

Infections are a common cause of morbidity and mortality after renal transplant (RT). The commonest cause for cryptococcal meningitis (CM) outside HIV infection is immunosuppression in RT patients, especially in cryptococcus-endemic regions [1].

Aetiology of infection varies with duration after transplant with the majority of systemic fungal infections occurring within 180 days after renal transplantation. Of those that occur after 180 days, the most common aetiologies are *Candida *and *Cryptococcus* [2]. Extra-pulmonary tuberculosis, including tuberculous meningitis (TBM), is one of the most common extra-pulmonary infections to occur post-RT [3]. Tuberculosis post-RT is associated with an increased risk for graft loss with higher mortality rates in those with disseminated rather than local disease [4].

CM and TBM account for 5-15% of infections in highly endemic areas and are associated with high mortality rates in renal transplant patients [2,5,6]. For tuberculous (TB) prevalence, Kenya is ranked no.15 out of 22 high-burden countries globally with population prevalence coming close to 1%. Approximately a third of HIV-infected patients are co-infected with CM in our setting [7]. There are case reports of concurrent fungal and TB infections outside the central nervous system (CNS) [8] post renal transplant, but none with simultaneous CM and TBM complicating renal transplant.

We report a unique case of successfully treated concurrent cryptococcal meningitis and tuberculous meningitis in a severely immunosuppressed renal transplant patient.

## Case presentation

A 67-year-old male with type 2 diabetes mellitus and hypertension for 18 years and resultant chronic kidney disease had a renal transplant three years prior and was immunosuppressed with prednisone 20mg OD, tacrolimus 0.3mg BD and mycophenolate 1g BD. He was referred to us with a three-week history of headaches, dizziness, unsteady gait, mild confusion, and seizures, as well as insidious weight loss and altered bowel habit for two months for which no cause had been found. He was HIV-negative. 

**Figure 1:**
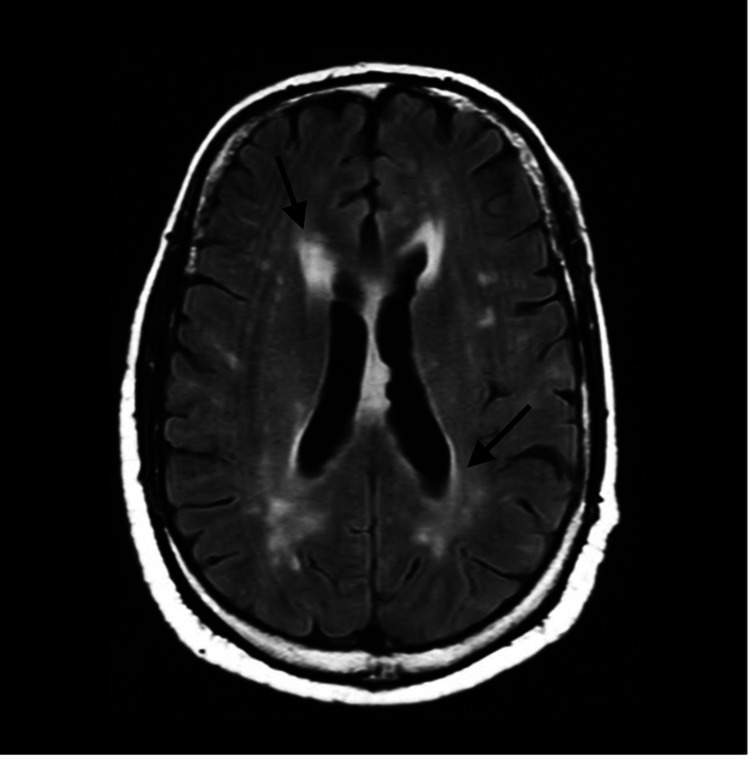
MRI of the brain on admission MRI: Magnetic resonance imaging Fluid-attenuated inversion recovery (FLAIR) sequences demonstrating confluent areas and foci of high signal in periventricular and deep white matter (black arrows).

Brain magnetic resonance imaging (MRI) revealed non-specific sulcal white matter hyperintensities (SWMHs) (Figure 1). Serology and cerebrospinal fluid (CSF) analysis confirmed cryptococcal meningitis with *Cryptococcus neoformans* grown on CSF cultures. He also had cytomegalovirus (CMV) viremia of log 4.67 but with no CMV in CSF. 

**Figure 2:**
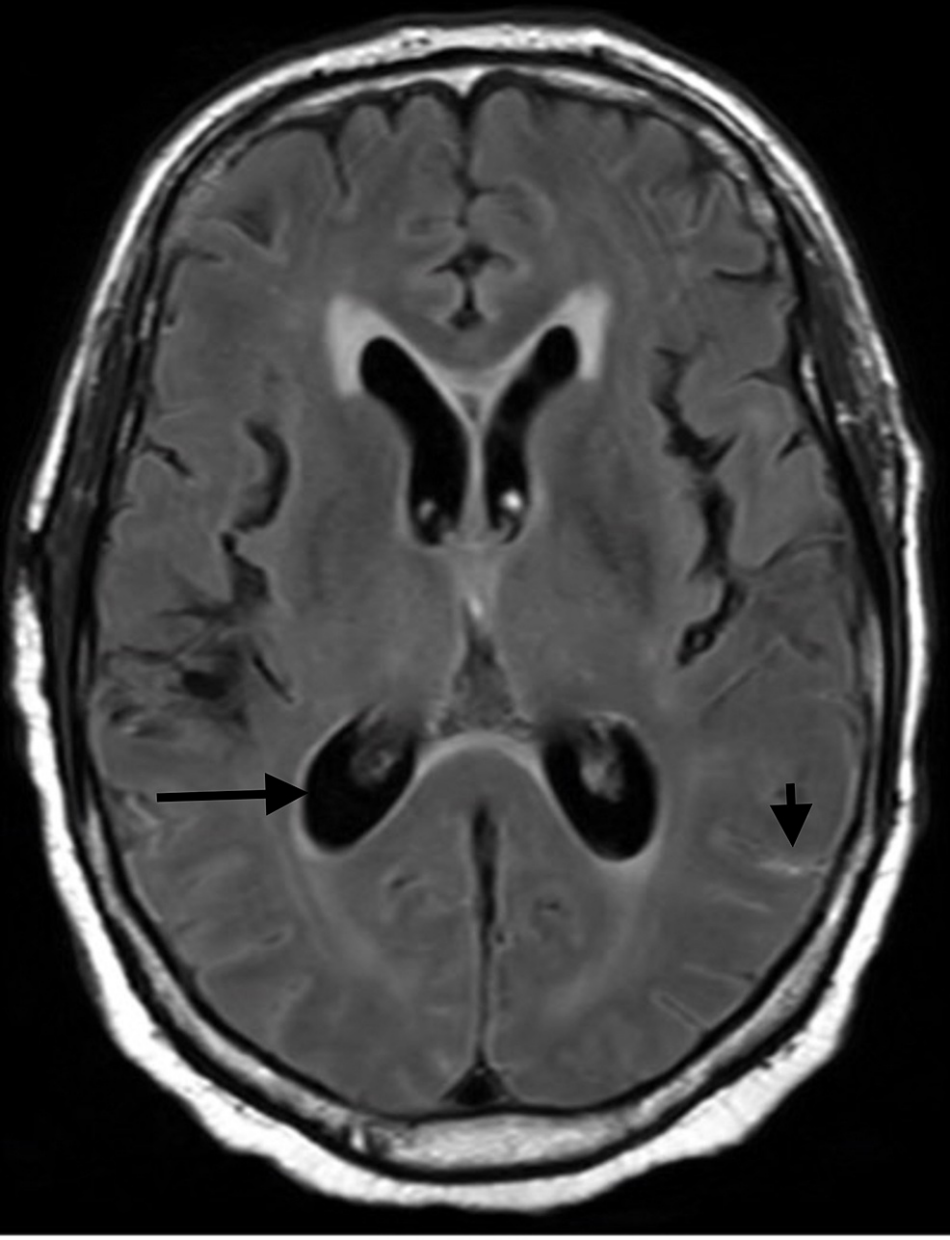
MRI of the brain on FLAIR sequences done three-weeks after admission The image is demonstrating new sulcal hyperintensities (short, black arrow) and hydrocephalus (long, black arrow) MRI: Magnetic Resonance Imaging, FLAIR: Fluid attenuated inversion recovery

**Figure 3:**
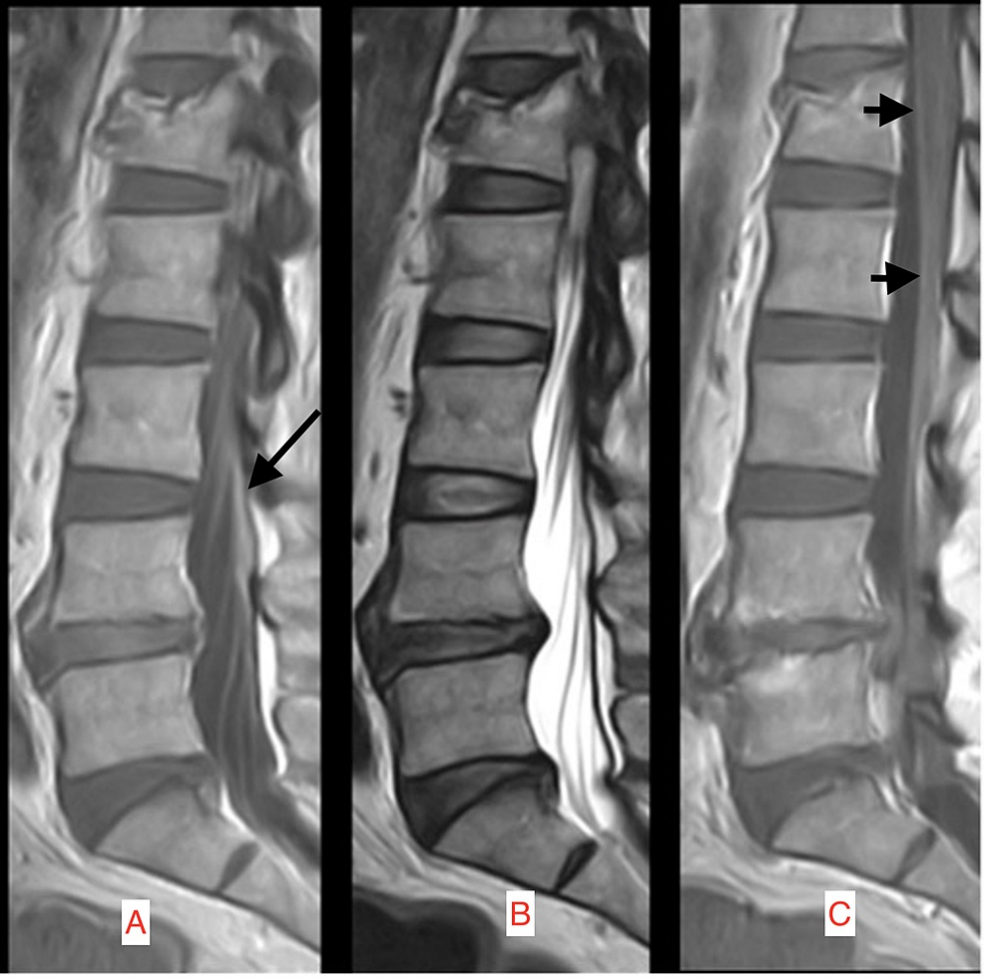
MRI of the spine done three weeks after admission The Magnetic Resonance Imaging (MRI) image is demonstrating a new enhancement of the distal spinal cord. A: Contrast-enhanced T1 sequence sagittal MRI image of the spine demonstrating cauda equina enhancement (long arrow). B: T2 sequence sagittal MRI image of the distal spinal cord corresponding to image A. C: Contrast-enhanced T1 sequence sagittal MRI image of the spine demonstrating lumbar spine enhancement (short arrows).

He initially improved after two weeks of liposomal amphotericin B and flucytosine, serial lumbar punctures to relieve raised intracranial pressure which eventually showed sterile CSF, and valganciclovir for gastrointestinal CMV. His immunosuppressive regimen was tapered down. However, he then deteriorated with reduced consciousness and uncontrollable seizures. Repeat MRI brain showed worsening SWMHs and interval development of hydrocephalus (Figure 2) and new meningeal enhancement of the distal spinal cord and cauda equina (Figure 3).

Repeat CSF examination revealed raised protein with leucocytosis, but repeat CSF TB cultures and polymerase chain reaction (PCR) test were both negative (see Table 1 for serial CSF analyses).

**Table 1:** Summary of serial CSF findings AAFB: Acid-Alcohol fast bacilli; CSF: Cerebro-spinal fluid; ATT: Antitubercular treatment; PCR: Polymerase chain reaction

CSF constituent	Units	Normal range	On admission	After 2 weeks	2 weeks of ATT	On discharge
White cell count	/mm3		<5	77 (89% lymphocytes)	19	
Red cell count	/mm3		<5	<5	50	
AAFB			Negative	Negative		
GeneXpert PCR			Negative	Negative		
Amino-deaminase levels	IU/L	6.5	<9.0			
Lactate	mmol/L	1.1-2.4	8.27			
Glucose	mmol/L	2.22-3.89	4.31	3.29	0.59	2.68
Protein	g/L	0.15-0.45	0.51	0.87	0.7	0.44
Cryptococccal antigen			Positive	Positive		
Culture			Cryptococcus			
Opening pressure	cm H20	<20	32	31	18	18

His case was discussed in the neurosciences multidisciplinary meeting and all imaging was reviewed from the admission. Although chest X-rays showed no lung pathology, CT coronary angiography done on admission had shown a right apical lesion with mediastinal lymphadenopathy. Further, the scout imaging on the repeat MRI scan had captured images of the chest, which showed worsening pulmonary nodules and moderate bilateral pleural effusions, most in keeping with TB. We, therefore, diagnosed TBM and commenced anti-tuberculous therapy with rifampicin, isoniazid, pyrazinamide, and ethambutol, as well as dexamethasone 8mg BD (twice a day). His neurologic status improved such that he was alert, orientated, and seizure-free when discharged home one month later.

## Discussion

Our patient was on three immunosuppressive agents which is more likely to predispose to opportunistic infections [8], and also had induction therapy with anti-thymocyte globulin which increases invasive fungal infection risk in renal transplant [2]. Other risk factors for infection in these patients include poor allograft function with frequent relapses and being a recipient of deceased donor kidney transplants due to a higher incidence of human leukocyte antigen (HLA) mismatch and more immunosuppression [9].

There may be minimal presenting signs and symptoms of infection as these may be masked by immunosuppression [10]. Presentation with headache and meningitis in CM is associated with prolonged (>30 days) hospital stay as was the case in our patient [1]. 

Diagnosis of extra-pulmonary TB in RT is usually by suggestive chest radiography rather than tissue culture [3], although in our case the anterior-posterior portable chest X-rays were not reliable and the diagnosis was made through a complete review of the other imaging modalities. Challenges in the diagnosis of mycobacterial infections in these patients may occur due to atypical presentation and this may result in treatment delays and poor outcomes [5]. Cultures and PCR can be negative in CNS TB [11] but do not preclude the diagnosis.

The lack of tissue or microbiological evidence of TB in our case was due to the challenges of diagnosing CNS TB. It is difficult to perform a biopsy on nervous system tissue, and CSF provides a very low yield in terms of diagnostic accuracy of TB [11]. In our practice and experience as an institution, we look very hard for evidence of TB, which eventually we did surmise from the neuro-imaging and chest imaging findings as discussed in the multidisciplinary team (MDT). TB is high up on the list of opportunistic infections, and we usually have a low threshold for treatment.

It is also possible that our patient may have had a paradoxical worsening of his symptoms due to immune reconstitution (IRIS). We have previously recognized and reported neurological deterioration due to IRIS in the context of CMV infection of the CNS in patients with HIV [12]. The complexity of neurological opportunistic infections in our settings has driven the need for our weekly neurosciences MDT meeting to come up with a consensus. The possibility of paradoxical worsening due to IRIS or similar pathophysiological mechanisms was discussed, but the constellation of the meningeal enhancement, hydrocephalus, worsening SMWHs, and chest findings pointed towards TB. 

It is recommended that all RT patients should have a pre-transplant assessment for latent TB infection with further confirmatory radiography before commencing immunosuppression [13], but this is not routine practice and was not done in our patient as he was transferred in from another TB-endemic country. Isoniazid prophylaxis may be offered to transplant patients with a history of inadequately treated TB or to those with a negative latent TB infection screen receiving a kidney from a donor with a positive latent TB screen [10].

At present, there is no recommendation for routine screening for cryptococcus or commencing primary antifungal prophylaxis in RT, but previous infections requiring enhanced immunosuppression may require secondary prophylaxis [14]. Treatment of cryptococcal meningitis post renal transplant involves an induction phase with liposomal amphotericin B and flucytosine for at least two weeks followed by eight weeks of fluconazole and then maintenance therapy with fluconazole for six months to one year [14]. The availability of the above induction agents may vary; however, deoxycholate amphotericin B is not recommended as the first-line due to the risk of nephrotoxicity [15].

Treatment of TB meningitis in these patients may involve a longer duration of treatment and closer follow-up due to challenges such as drug interactions, drug adverse effects, and preservation of renal graft function [16]. Renal function needs to be monitored closely as TB post-transplantation is associated with higher risks of acute kidney injury with a possibility of not returning to baseline renal function [17].

Treatment of opportunistic infections in these patients involves a stepwise reduction in immunosuppression [15]. This may predispose to immune reconstitution syndrome which may result in paradoxical worsening of symptoms [14].

## Conclusions

This case is, to our knowledge, the first case report of concurrent cryptococcal and tuberculous meningitis in a post-renal transplant patient. It highlights the need to thoroughly investigate for a concurrent CNS opportunistic infection in patients post renal transplant who fail to improve after treatment for the first identified infection. The case also outlines the challenges in the management of multiple opportunistic infections while maintaining graft function.
